# The effect of STAT1, miR-99b, and MAP2K1 in alcoholic liver disease (ALD) mouse model and hepatocyte

**DOI:** 10.18632/aging.205579

**Published:** 2024-02-29

**Authors:** Hongbo Du, Hao Yu, Meiyue Zhou, Quan Hui, Yixin Hou, Yuyong Jiang

**Affiliations:** 1 Beijing Ditan Hospital Capital Medical University, Beijing 100015, China; 2 Dongzhimen Hospital, Beijing University of Chinese Medicine, Beijing 100015, China

**Keywords:** ALD, miR-99b, STAT1, MAP2K1

## Abstract

Alcoholic liver disease (ALD) serves as the leading cause of chronic liver diseases-related morbidity and mortality, which threatens the life of millions of patients in the world. However, the molecular mechanisms underlying ALD progression remain unclear. Here, we applied microarray analysis and experimental approaches to identify miRNAs and related regulatory signaling that associated with ALD. Microarray analysis identified that the expression of miR-99b was elevated in the ALD mouse model. The AML-12 cells were treated with EtOH and the expression of miR-99b was enhanced in the cells. The expression of miR-99b was positively correlated with ALT levels in the ALD mice. The microarray analysis identified the abnormally expressed mRNAs in ALD mice and the overlap analysis was performed with based on the differently expressed mRNAs and the transcriptional factors of miR-99b, in which STAT1 was identified. The elevated expression of STAT1 was validated in ALD mice. Meanwhile, the treatment of EtOH induced the expression of STAT1 in the AML-12 cells. The expression of STAT1 was positively correlated with ALT levels in the ALD mice. The positive correlation of STAT1 and miR-99b expression was identified in bioinformatics analysis and ALD mice. The expression of miR-99b and pri-miR-99b was promoted by the overexpression of STAT1 in AML-12 cells. ChIP analysis confirmed the enrichment of STAT1 on miR-99b promoter in AML-12 cells. Next, we found that the expression of mitogen-activated protein kinase kinase 1 (MAP2K1) was negatively associated with miR-99b. The expression of MAP2K1 was downregulated in ALD mice. Consistently, the expression of MAP2K1 was reduced by the treatment of EtOH in AML-12 cells. The expression of MAP2K1 was negative correlated with ALT levels in the ALD mice. We identified the binding site of MAP2K1 and miR-99b. Meanwhile, the treatment of miR-99b mimic repressed the luciferase activity of MAP2K1 in AML-12 cells. The expression of MAP2K1 was suppressed by miR-99b in the cells. We observed that the expression of MAP2K1 was inhibited by the overexpression of STAT1 in AML-12 cells. Meanwhile, the apoptosis of AML-12 cells was induced by the treatment of EtOH, while miR-99b mimic promoted but the overexpression of MAP2K1 attenuated the effect of EtOH in the cells. In conclusion, we identified the correlation and effect of STAT1, miR-99b, and MAP2K1 in ALD mouse model and hepatocyte. STAT1, miR-99b, and MAP2K1 may serve as potential therapeutic target of ALD.

## INTRODUCTION

Alcoholic liver disease (ALD) is the leading cause of chronic liver diseases-related morbidity and mortality, which threatens the life of millions of patients globally every year [1, 2]. ALD pathogenesis is complex and multifactorial, and commonly progresses through several phases namely the alcoholic steatosis, hepatitis, fibrosis, and cirrhosis [3]. ALD is characterized by excessive triglycerides deposition in the hepatocytes at early stage, and further inflammatory response and oxidative stress, which promoted steatohepatitis [4]. Noteworthy, it has been suggested that ethanol stimulates metabolic pressure and the oxidative stress during pathological processes of liver [5]. Moreover, increasing evidences have confirmed the pivotal role of inflammation during liver injury, including the ALD [6]. Despite of the great progression that has been made in the past years, the specific molecular and cellular mechanisms underlying the pathogenesis of ALD has not yet been fully completed, and further understanding is an urgent for exploration of advantageous therapeutic targets [7].

Large-scale gene expression analysis such as microarray analysis has made great contribution in identifying specific and critical genes that modulate disease progression, which facilitates the quick progression on drug development [8–10]. Over the past decade, microarray technology has been widely applied in profiling the differentially expressed genes in liver diseases including the ALD [11, 12]. For example, a transcriptional and immune profiling of non-alcoholic steatohepatitis (NASH) samples indicated that NASH is associated with elevated CD8 T cells in liver and altered antigen-presenting and cytotoxic cells in blood [13]. A recent microarray data analysis identified 4 non-alcoholic fatty liver disease (NAFLD)-related hub genes (DUSP1, NR4A1, FOSB, ZFP36) as diagnostic markers in chronic kidney disease patients with NAFLD [14]. However, the microarray analysis on differentially expressed genes and microRNAs (miRNAs) are limited.

MiRNAs are non-coding RNAs with short sequences, which modulate gene expression via interacting with the mRNAs to wither suppress their translation or promote RNA degradation [15]. Accumulating evidence have exposed the important role of miRNAs in ALD progression [16–18]. For example, a miRNA profile indicated that miR-214, miR-203 and miR-539 were abnormally expressed in liver samples from patients with alcoholic steatohepatitis (ASH) [18].

In this work, we adopted informatic analysis and experimental approaches to identify miRNAs and related regulatory signaling that associated with ALD. We identified a notable elevation of miR-99b in mouse ALD model, which is correlated with elevated level of alanine aminotransferase (ALT). Further analysis on upstream and downstream factors correlated with miR-99b indicated a STAT1/miR-99b/mitogen-activated protein kinase kinase 1 (MAP2K1) regulatory signaling. Our work may provide a novel therapeutic approach for ALD therapy.

## MATERIALS AND METHODS

### Animal model

All experiments were approved by the Animal Ethic Committee of Dongzhimen Hospital, Beijing University of Chinese Medicine. Female C57BL/6 mouse aged 6-weeks old were purchased from The Jackson Laboratory (USA). To establish ALD, mouse received a Lieber-DeCarli diet that contains 5% (v/v) alcohol or an isocaloric dextrin maltose diet for three months as per previous report [12]. The use of animals has ethical approval in addition to following guidelines All animal experiments were performed in compliance with Animal Use and Care Committee directives of Dongzhimen Hospital, Beijing University of Chinese Medicine. After treatment, the mice were sacrificed, liver samples and serum were collected for subsequent study. The serum concentration of alanine aminotransferase (ALT) and aspartate transaminase (AST) was detected using CheKine™ Alanine Aminotransferase (ALT/GPT) Activity Colorimetric Assay Kit (Abbkine, China) and Aspartate Aminotransferase Activity Assay kit (Abcam, USA) in line with manufacture’s description, respectively.

### Microarray analysis

Total RNA was extracted from the liver tissues that collected from ALD mouse and control mouse by using the TRIzol reagent (Invitrogen, USA) according to the manufacturer’s description. The RNA was then quantified and approximately 5 μg of total RNA was used to construct cDNA library following a standard protocol. The differentially expressed miRNAs (DEmi) and differentially expressed genes (DEGs) were determined by using the “limma” package in R software (version 4.0.0). The |log2 fold change (FC)| > 0.5 and *P*-value < 0.05 were set as the cut-off criteria to determine DEmis and DEGs. The WGCNA R package was used to cluster the DEmis into differently colored modules using the dissimilarity measure (1-topological overlap measure (TOM), TOM ≥ 0.15) [19].

### Cell line and treatment

The mouse hepatocyte cell line Alpha mouse liver (AML)-12 was brought from American Type Culture Collection (ATCC, USA), and maintained in DMEM/F-12 medium (Hyclone, USA), that contains 10% (v/v) fetal bovine serum (Life technologies, 10099-141), 5 mg/ml insulin (Sigma-Aldrich, I9278), 5 μg/ml transferrin (Sigma-Aldrich, USA), 5 ng/ml selenium (Sigma-Aldrich, USA), 40 ng/ml dexamethasone (Sigma-Aldrich, USA), 100 U/ml penicillin, and 100 μg/ml streptomycin (Sigma-Aldrich, USA) at 37°C humidified atmosphere with 5% CO_2_. To establish ALD cell model, AML-12 cells were treated with 100 mM EtOH for 48 hours, and collected for further experiments.

### Cell transfection

The sequence of STAT1 and MAP2K1 CDSs were obtained from NCBI database and synthesized by GenePharma (Shanghai, China). The overexpressing vectors of MAP2K1 (MAP2K1 OE) and STAT1 (STAT1 OE) were constructed by inserting the CDS sequences of targeted genes into the pcDNA 3.1 vectors. The miR-99b mimics and negative control [20] mimics were synthesized by GenePharma. Transient cells transfection was performed by using the Lipofectamine 2000 according to the manufacturer’s instructions.

### Cell apoptosis

Cell apoptosis was assessed by the Annexin V/PI apoptosis detection kit (Beyotime, China). In short, cells were collected and suspended in binding buffer, stained with Annexin V (5 μg/ml) and PI (5 μg/ml) in dark for 30 minutes. The cells were then analyzed by using flow cytometry (BD Biosciences, USA).

### Quantitative real-time PCR

The total RNA extraction from liver tissues and cells were conducted by using the Trizol reagent (Invitrogen, USA). The RNA was then quantified, reverse transcribed to cDNA using a First strand synthesis kit (Transgen, China). The real time PCR was performed to quantify the gene levels using the SYBR Green PCR Master Mix (Takara, China). Relative gene expression was determined by normalization to U6 or Tublin.

Primers: pri-miR-99b: F: 5′-ctcctgggtcctggcacccac-3′; R: 5′-cccgacacggacccatagaca-3′; miR-99b: F: 5′-cacccgtagaaccgac-3′; R: 5′-GTGCAGGGTCCGAGGT-3′; U6: F:5′-CTCGCTTCGGCACATA-3′; R:5′-CGAATTTGCGTGTCATCCT-3′; STAT1: F: 5′-TCACAGTGGTTCGAGCTTCAG-3′; R:5′-GCAAACGAGACATCATAGGCA-3′; MAP2K1: F: 5′-AAGGTGGGGGAACTGAAGGAT-3′; R: 5′-CGGATTGCGGGTTTGATCTC-3′.

### Western blotting

Cells were collected and were lysed in an ice-cold RIPA lysis buffer (Thermo, USA). The total proteins were quantified using a BCA Protein Assay Kit (Beyotime, China). Equal amounts of proteins (40 μg) were separated on an 8 to 12% SDS-PAGE gel and shifted to nitrocellulose [20] membrane. The membranes were blocked with 5% skim milk for 2 hours and were probed with primary antibodies against Stat1 (1;1000, Abcam, USA), MAP2K1 (1;1000, Abcam, USA), Tubulin (1;1000, Abcam, USA) at 4°C overnight. The protein bands were then hatched with horseradish peroxidase (HRP)-conjugated IgG secondary antibodies, and visualized by enhanced chemiluminescence (ECL, Millipore, USA).

### Luciferase reporter gene assay

The sequence of the MAP2K1 3’UTR region was cloned into the pmirGLO vectors (Promega, USA) to generate MAP2K1-WT vectors. The site-specific mutation was performed to obtain MAP2K1-mut vectors. Cells were co-transfected with miR-99b-5p mimics or NC mimics and MAP2K1-WT or MAP2K1-mut for 36 hours, and harvested to test the luciferase activity by using a dual luciferase reporter gene assay kit (Promega, USA). The pRLTK renilla luciferase reporter vector (Promega, USA) was adopted as the internal control.

### Chromatin immunoprecipitation (ChIP) assay

ChIP assay was performed by using the EZ-ChIP kit (Millipore, USA) following the manufacturer’s instruction. In short, cells were fixed with 1% formaldehyde for 10 min to generate DNA-protein crosslinks and homogenized to obtain 800 μl of cell lysates. The cell lysates were sonicated to generate short chromatin fragments of 200 to 500 bp. Half of the lysates were stored as input. And 100 μl of cell lysates were immunoprecipitated with 1 μl of STAT1 antibody or IgG as control. The precipitation was then subjected to qRT-PCR assay to analyze the level of miR-99b.

### Statistical analysis

All data were presented as mean ± SD for at least three independent tests. Statistical differences between two or multiple groups were evaluated using a student’s *t* test or one-way analysis of variance (ANOVA). Differences were regarded as statistically significant when *p* < 0.05.

## RESULTS

### The upregulation of miR-99b is identified by microarray analysis in ALD mouse model

**Figure 1:**
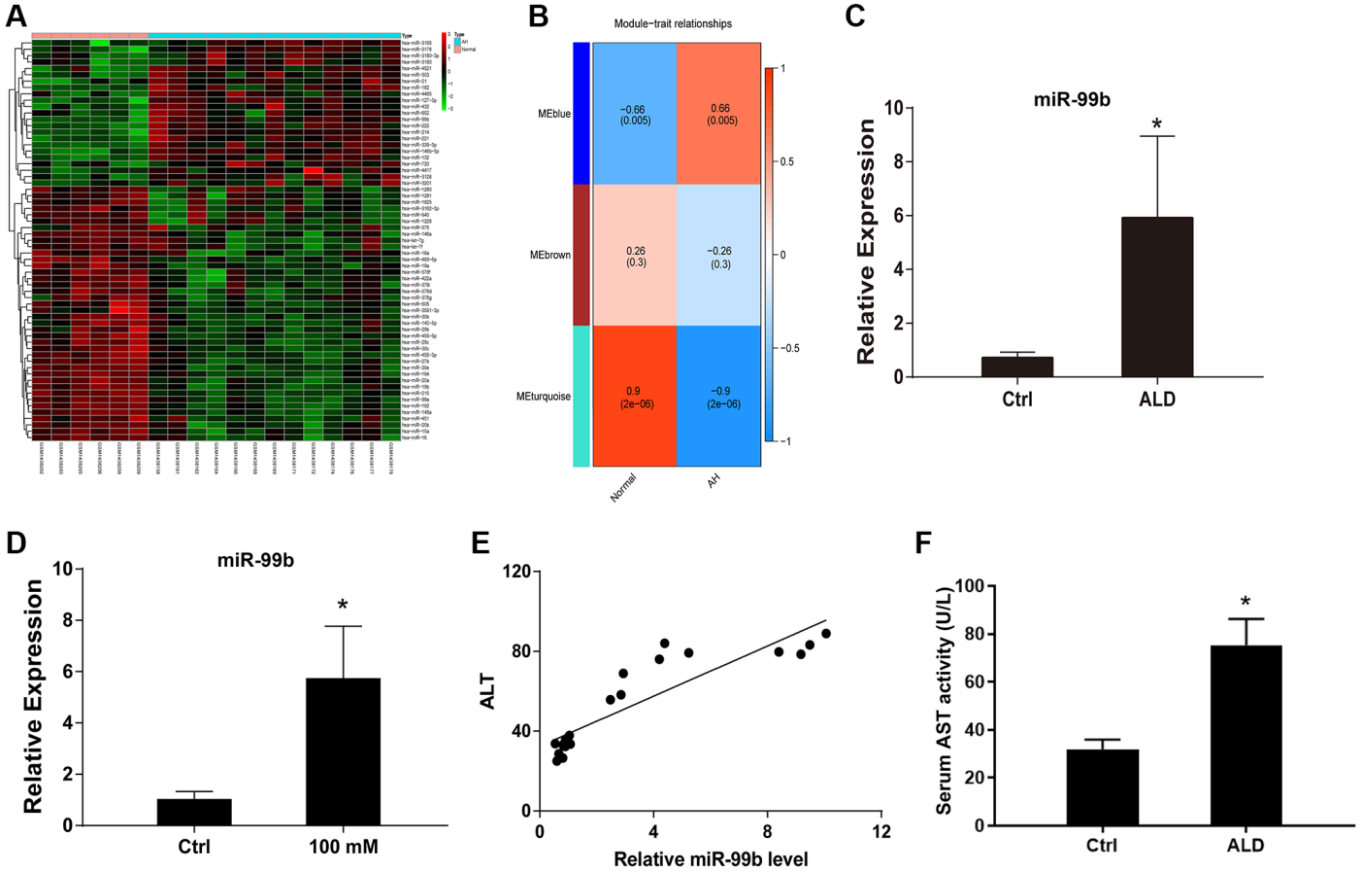
**The upregulation of miR-99b is identified by microarray analysis in ALD mouse model.** (**A**, **B**) The microarray analysis was performed in ALD mice. The miRNA heatmap (**A**) and WGCNA (**B**) were shown. (**C**) The expression of miR-99b was detected by qPCR in ALD mice. (**D**) The expression of miR-99b was measured by qPCR in AML-12 cells treated with 100 mM EtOH. (**E**) The correlation of miR-99b with ALT levels was analyzed in ALD mice. (**F**) The serum AST level. ^*^P < 0.05.

To explore the abnormally expressed miRNAs in ALD, we performed microarray analysis in ALD mouse model. And multiple miRNAs were identified in the model (Figure 1A, 1B). In addition, we identified that the expression of miR-99b was elevated in the ALD mouse model (Figure 1C). Meanwhile, the AML-12 cells were treated with EtOH and the expression of miR-99b was enhanced in the cells (Figure 1D). Significantly, the expression of miR-99b was positively correlated with ALT levels in the ALD mice (Figure 1E). The elevated level of AST confirmed the liver damage and successful establishment of ALD model (Figure 1F).

### STAT1 is found as an upstream factor of miR-99b by microarray analysis in ALD mouse model

**Figure 2:**
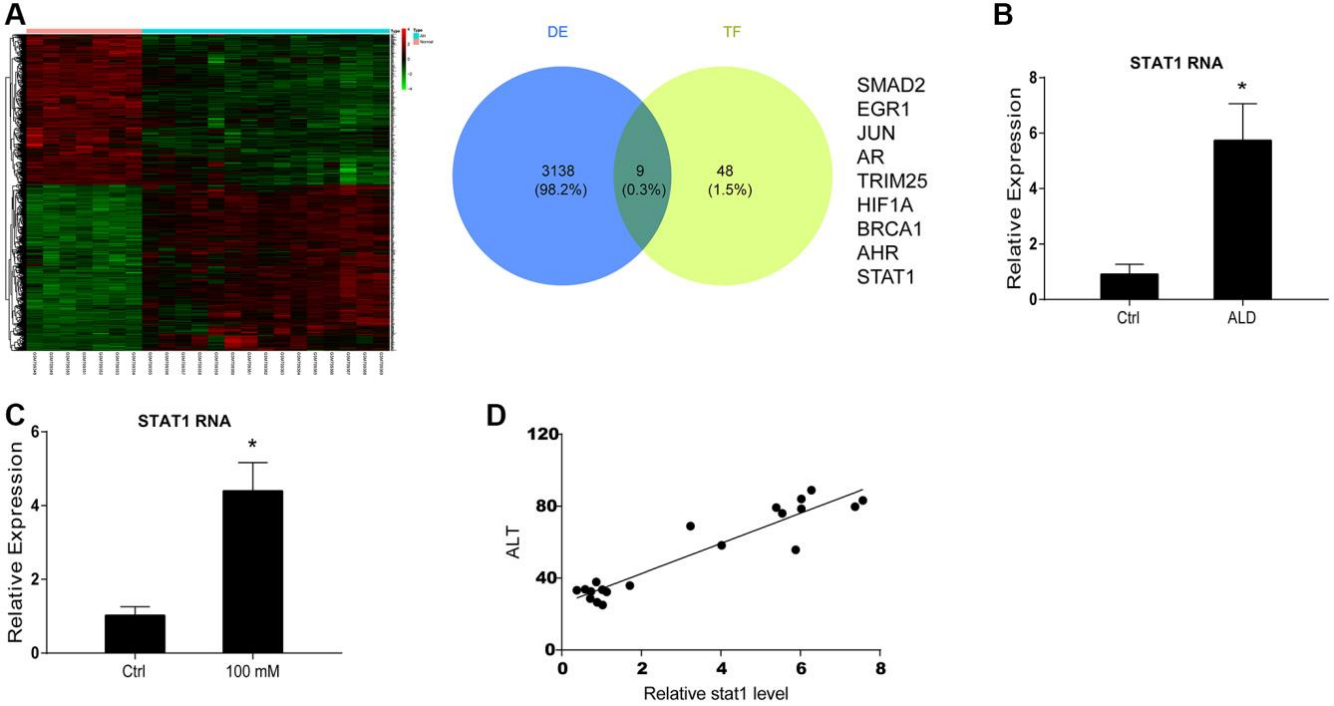
**STAT1 is found as an upstream factor of miR-99b by microarray analysis in ALD mouse model.** (**A**) The microarray analysis was performed in ALD mice. The mRNA heatmap was shown. The overlap was performed using microarray analysis and TransmiR v2.0 database. (**B**) The expression of STAT1 was measured by qPCR in ALD mice. (**C**) The expression of STAT1 was measured by qPCR in AML-12 cells treated with 100 mM EtOH. (**D**) The correlation of STAT1 with ALT levels was analyzed in ALD mice. ^*^P < 0.05.

Next, we tried to identify the upstream factors of miR-99b in ALD. The microarray analysis identified the abnormally expressed mRNAs in ALD mice and the overlap analysis was performed based on the differently expressed mRNAs and the transcriptional factors of miR-99b, in which STAT1 was identified (Figure 2A). We next validated these findings using *in vitro* and *in vivo* models. The elevated expression of STAT1 was validated in ALD mice (Figure 2B). Meanwhile, the treatment of EtOH induced the expression of STAT1 in the AML-12 cells (Figure 2C). The expression of STAT1 was positively correlated with ALT levels in the ALD mice (Figure 2D). These data indicated that miR-99b and STAT1 potentially participate in the ALD generation.

### STAT1 enhances miR-99b expression in ALD model

**Figure 3:**
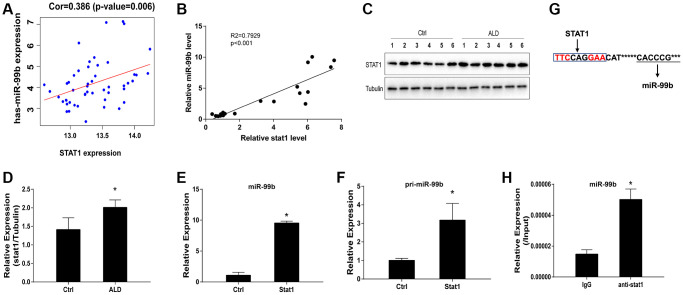
**STAT1 enhances miR-99b expression in ALD model.** (**A**) The correlation of STAT1 and miR-99b was analyzed using bioinformatic analysis. (**B**) The correlation of STAT1 and miR-99b was measured by qPCR in ALD mice. (**C**, **D**) The expression of STAT1 was detected by Western blot analysis in ALD mice. (**E**, **F**) The AML-12 cells were transfected with STAT1 overexpressing plasmid and the expression of miR-99b (**E**) and pri-miR-99b (**F**) was determined in the cells. (**G**) Prediction of STAT1 binding site on miR-99b promoter region. (**H**) The enrichment of STAT1 on miR-99b promoter was identified by ChIP analysis in AML-12 cells. ^*^P < 0.05.

The positive correlation of STAT1 and miR-99b expression was identified by the bioinformatics analysis (Figure 3A). Consistently, we then performed qPCR assay and identified this positive correlation (Figure 3B). Meanwhile, the protein levels of STAT1 were upregulated in ALD mice (Figure 3C, 3D). We next performed STAT1 overexpression *in vitro* to assess the corresponding alteration of miR-99b. Overexpression of STAT1 successfully upregulated the STAT1 expression in hepatocytes (Supplementary Figure 1A). Significantly, the expression of miR-99b (Figure 3E) and pri-miR-99b (Figure 3F) was promoted by the overexpression of STAT1 in AML-12 cells. Moreover, previous study indicated that STAT1 usually interact with the gamma-activated site (GAS) region [21], which is content on the upstream of miR-99b. Prediction of STAT1 binding site on miR-99b promoter region was shown (Figure 3G). The Further ChIP analysis confirmed the enrichment of STAT1 on miR-99b promoter in AML-12 cells (Figure 3H).

### MAP2K1 is negatively correlated with miR-99b and downregulated in ALD model

**Figure 4:**
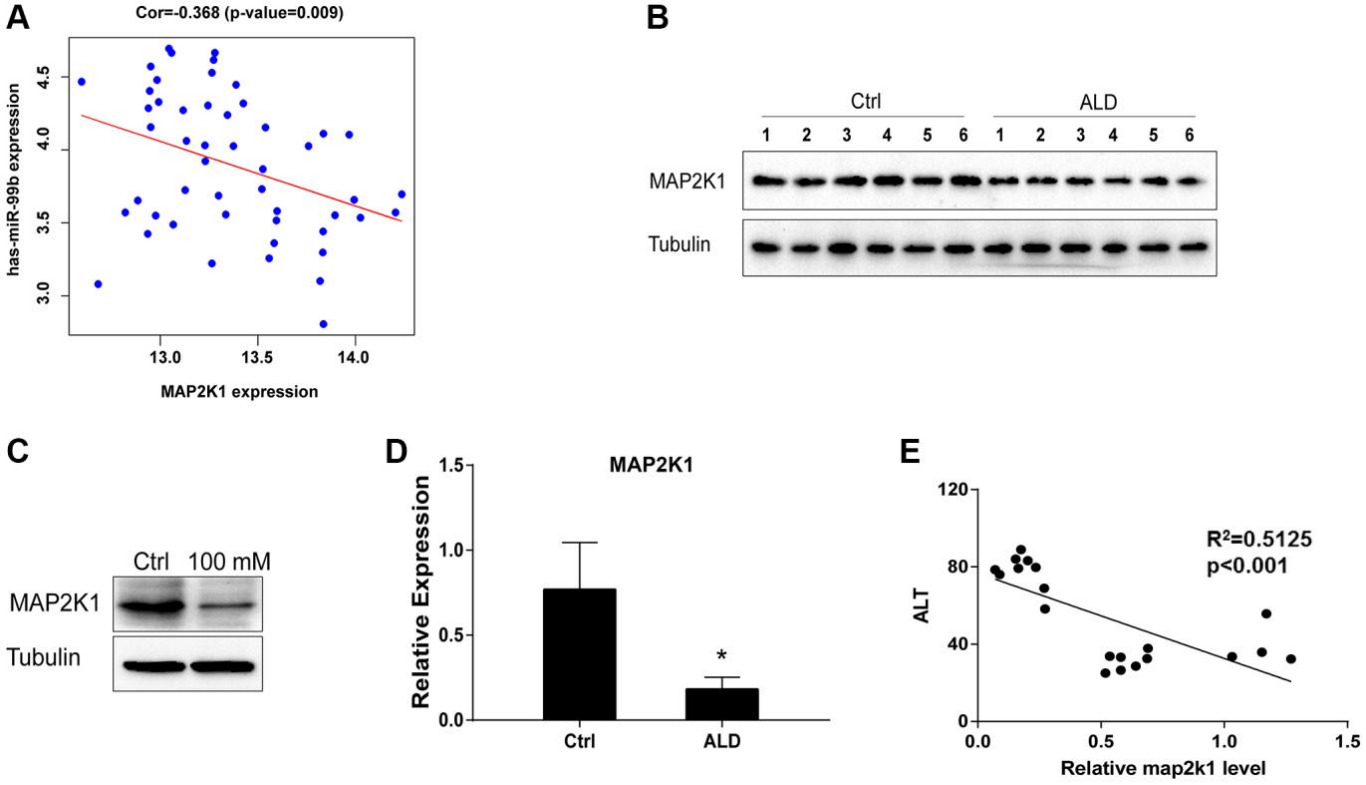
**MAP2K1 is negatively correlated with miR-99b and downregulated in ALD model.** (**A**) The correlation of MAP2K1 and miR-99b was analyzed using bioinformatic analysis. (**B**) The expression of MAP2K1 was detected by Western blot analysis in ALD mice. (**C**) The expression of MAP2K1 was detected by qPCR in ALD mice. (**D**) The expression of MAP2K1 was measured by qPCR in AML-12 cells treated with 100 mM EtOH. (**E**) The correlation of MAP2K1 with ALT levels was analyzed in ALD mice. ^*^P < 0.05.

Next, the miRWalk database predicted the target genes of miR-99b and then intersected with the genes downregulated in the mRNA microarray, in which 32 genes were negatively correlated with miR-99b in the bioinformatic analysis using TCGA database, including MAP2K1 and GADD45G. We validated that the expression of MAP2K1 was negatively associated with miR-99b (Figure 4A). The expression of MAP2K1 was downregulated in ALD mice (Figure 4B, 4C). Consistently, the expression of MAP2K1 was reduced by the treatment of EtOH in AML-12 cells (Figure 4D). The expression of MAP2K1 was negative correlated with ALT levels in the ALD mice (Figure 4E).

### miR-99b targets MAP2K1 in AML-12 cells

**Figure 5:**
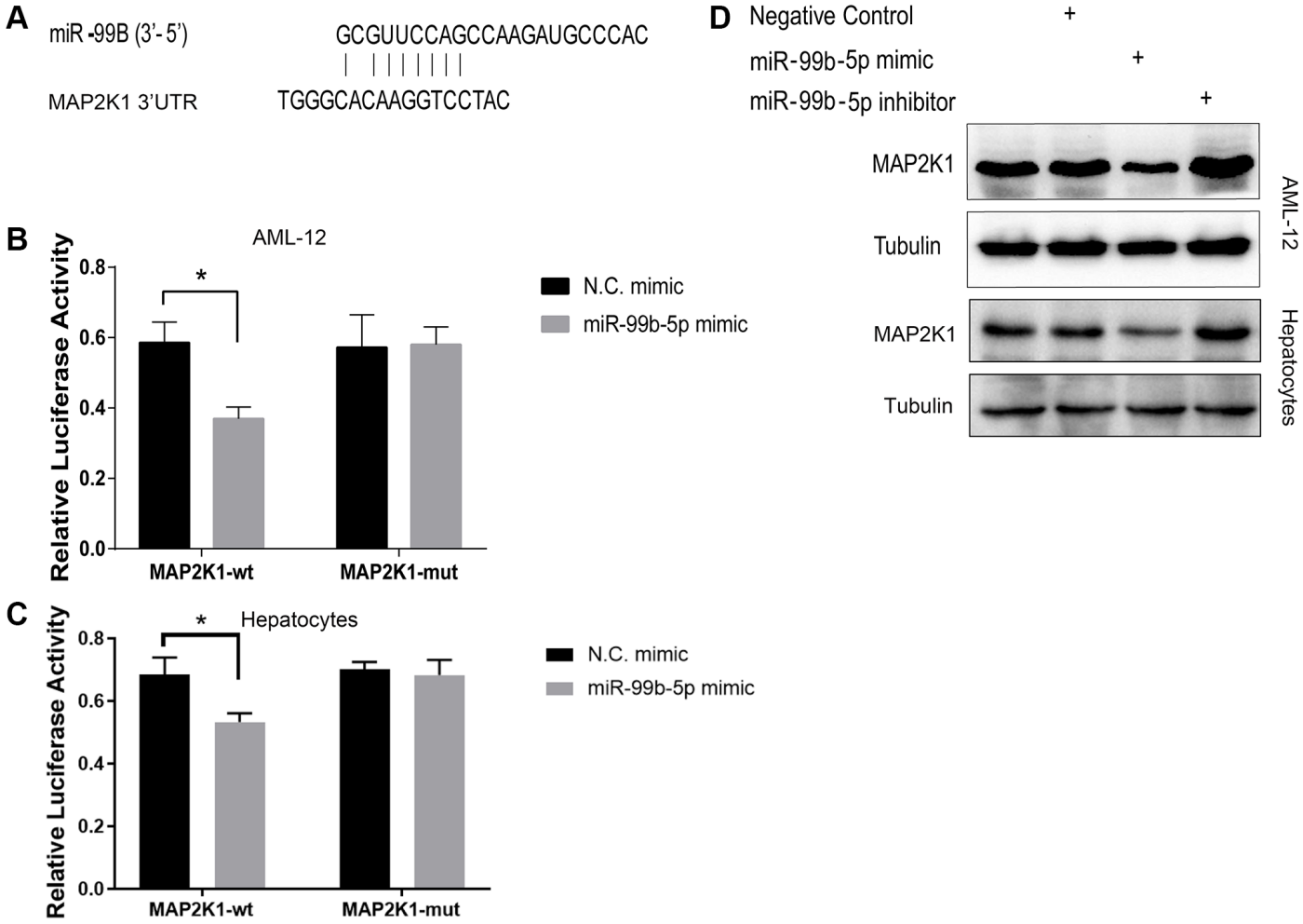
**mir-99b targets MAP2K1 in AML-12 cells.** (**A**) The binding analysis between MAP2K1 and miR-99b. (**B**, **C**) The dual luciferase reporter assays of MAP2K1 were conducted in (**B**) AML-12 cells and (**C**) primary hepatocytes treated with control mimic or miR-99b mimic. (**D**) The Western blot analysis of MAP2K1 was carried out in the AML-12 cells treated with control mimic or miR-99b mimic. ^*^P < 0.05.

Then, we identified the binding site of MAP2K1 and miR-99b (Figure 5A). Meanwhile, the treatment of miR-99b mimic repressed the luciferase activity of MAP2K1 in AML-12 cells (Figure 5B) and primary hepatocytes (Figure 5C). The expression of MAP2K1 was suppressed by miR-99b in AML-12 cells and primary hepatocytes (Figure 5D).

### STAT1/miR-99b/MAP2K1 axis regulates apoptosis of AML-12 cells

**Figure 6:**
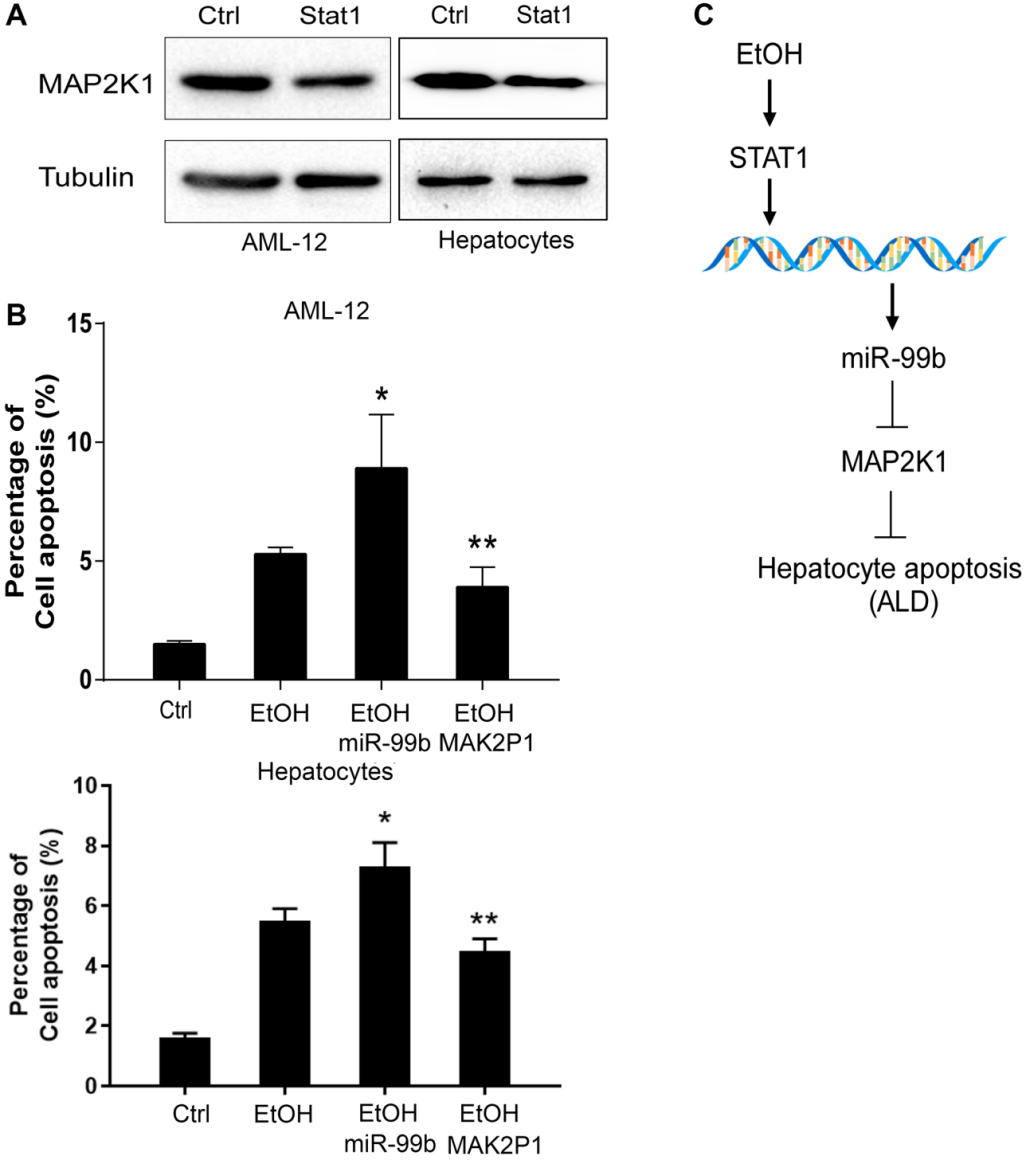
**STAT1/miR-99b/MAP2K1 axis regulates apoptosis of AML-12 cells.** (**A**) The AML-12 cells were transfected with STAT1 overexpressing plasmid and the expression of MAP2K1 was determined in the cells. (**B**) The apoptosis was analyzed in AML-12 cells treated with EtOH, or cotreated with EtOH and miR-99b mimic or MAP2K1 overexpressing plasmid. (**C**) A hypothesis model of this study was shown. ^*^P < 0.05, ^**^P < 0.01.

Next, we investigated the STAT1/miR-99b/MAP2K1 axis in hepatocytes. We observed that the expression of MAP2K1 was inhibited by the overexpression of STAT1 in AML-12 cells (Figure 6A). The apoptosis of AML-12 cells was induced by the treatment of EtOH, and miR-99b mimic promoted these effects. Meanwhile, the MAP2K1 overexpression vectors effectively elevated the expression of MAP2K1 expression (Supplementary Figure 1B) and abolished the promoting effects of miR-99b mimics on cell apoptosis (Figure 6B). The hypothesis model of this study was shown (Figure 6C).

## DISCUSSION

ALD serves as the leading cause of chronic liver diseases-related morbidity and mortality, which threatens the life of millions of patients in the world. Nevertheless, the molecular mechanisms underlying ALD progression are still elusive. In the current study, we used microarray analysis and experimental approaches to identify miRNAs and related regulatory signaling that associated with ALD.

MiRNAs play crucial effect in ALD progression. It has been reported that microRNA-200a promotes apoptosis by regulating ZEB2 in ALD [22]. MicroRNA-223 attenuates liver injury by repressing the IL-6-p47/phox-oxidative stress signaling in ALD [23]. Autophagy and lysosome are associated with exosome production by micro-RNA 155 in ALD [24]. In this study, microarray analysis identified that the expression of miR-99b was elevated in the ALD mouse model. The AML-12 cells were treated with EtOH and the expression of miR-99b was enhanced in the cells. The expression of miR-99b was positively correlated with ALT levels in the ALD mice. Our data demonstrated that miR-99b is associated with ALD *in vitro* and *in vivo*. The effect of miR-99b on ALD progression should be explored by more experiments in the future.

Furthermore, it has been found that NIK targeting PPARα by MAP2K1 disrupts hepatic fatty acid oxidation and exhibits high value in ALD therapy [25]. Rilpivirine represses liver fibrosis by selective STAT1-regulated apoptosis in hepatic stellate cells [20]. In this work, we demonstrated that the microarray analysis identified the abnormally expressed mRNAs in ALD mice, and the overlap analysis was performed with the differently expressed mRNAs and the transcriptional factors of miR-99b, in which STAT1 was identified. The elevated expression of STAT1 was validated in ALD mice from our model. Meanwhile, the treatment of EtOH induced the expression of STAT1 in the AML-12 cells. The expression of STAT1 was positively correlated with ALT levels in the ALD mice. The positive correlation of STAT1 and miR-99b expression was identified in bioinformatics analysis and validation in ALD mice by qPCR assay. We observed that the expression of miR-99b and pri-miR-99b was promoted by the overexpression of STAT1 in AML-12 cells. Further ChIP analysis confirmed the enrichment of STAT1 on miR-99b promoter in AML-12 cells.

Next, the miRWalk database predicted the target genes of miR-99b and then intersected with the genes downregulated in the mRNA microarray, in which 32 genes were negatively correlated with miR-99b in the bioinformatic analysis using TCGA database, including MAP2K1. The expression of MAP2K1 was negatively associated with miR-99b according to the microarray analysis.

MAP2K1, also known as MEK1, belongs to the mitogen-extracellular activated protein kinase kinase (MEK) family and is widely involved in cell survival and proliferation [26, 27]. Mutation and aberrant function have been implied in multiple diseases, especially cancers. Besides, MAP2K1 mutation activates RAS/MAPK signaling in endothelial cells and is associated with extracranial arteriovenous malformation [28]. MEK mediates the ERK signaling transduction to affect autophagy and consequently participates in the pathogenesis of liver diseases such as non-alcoholic fatty liver, liver fibrosis and hepatocellular carcinoma [29]. Noteworthy, the expression of MAP2K1 was confirmed downregulated in our ALD model. Consistently, the expression of MAP2K1 was reduced by the treatment of EtOH in AML-12 cells. The expression of MAP2K1 was negative correlated with ALT levels in the ALD mice. We identified the binding site of MAP2K1 and miR-99b. Meanwhile, the treatment of miR-99b mimic repressed the luciferase activity of MAP2K1 in AML-12 cells. The expression of MAP2K1 was suppressed by miR-99b in the cells. We observed that the expression of MAP2K1 was inhibited by the overexpression of STAT1 in AML-12 cells. Meanwhile, the apoptosis of AML-12 cells was induced by the treatment of EtOH, while miR-99b mimic promoted but the overexpression of MAP2K1 attenuated the effect of EtOH in the cells. These data indicate that STAT1, miR-99b, and MAP2K1 may play crucial role in the regulation of ALD and the effect of STAT1/miR-99b/MAP2K1 on ALD should be confirmed in future investigations.

In summary, we aimed to explore the potential regulatory mechanisms and therapeutic targets involved in alcoholic liver disease. We established ALD mouse model and performed microarray analysis, followed by experiments verification. We identified the correlation and effect of STAT1, miR-99b, and MAP2K1 in ALD mouse model and hepatocyte. STAT1, miR-99b, and MAP2K1 may serve as potential therapeutic target of ALD.

## Supplementary Materials

Supplementary Figure 1
