# Supplementary material for: The effect of STAT1, miR-99b, and MAP2K1 in alcoholic liver disease (ALD) mouse model and hepatocyte
Source: Aging (Albany NY). 2024 Feb 29;16(5):4224–35. doi: 10.18632/aging.205579 (PMC10968706; doi:10.18632/aging.205579)
Supplement: Supplementary Figure 1 [file aging-16-205579-s001.pdf]

SUPPLEMENTARY FIGURE

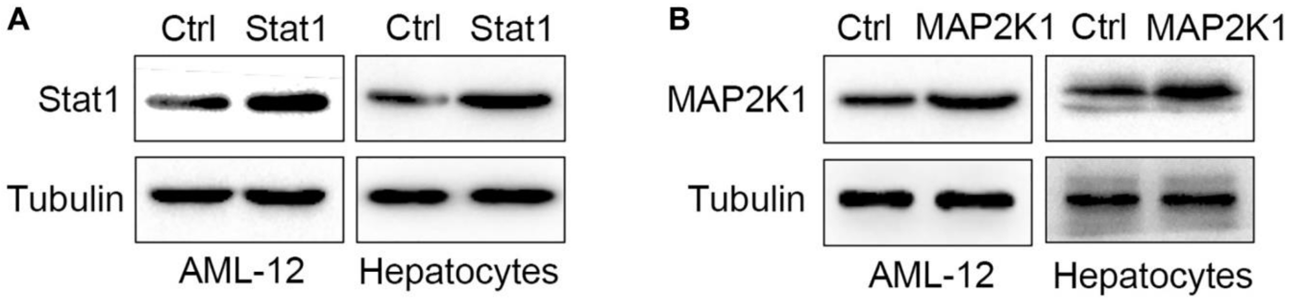

Supplementary Figure 1. (**A**) Expression of STAT1 in AML-12 cells and hepatocytes that transfected with STAT1 overexpression vectors. (**B**) Expression of MAP2K1 in AML-12 cells and hepatocytes that transfected with MAP2K1 overexpression vectors.
